# Innovations in Infant Feeding: Future Challenges and Opportunities in Obesity and Cardiometabolic Disease

**DOI:** 10.3390/nu12113508

**Published:** 2020-11-14

**Authors:** Julio Alvarez-Pitti, Ana de Blas, Empar Lurbe

**Affiliations:** 1 Department of Pediatrics, Consorcio Hospital General, University of Valencia, 46014 Valencia, Spain; adeblaszapata@gmail.com (A.d.B.); Empar.Lurbe@uv.es (E.L.); 2 CIBER Fisiopatología Obesidad y Nutrición (CB06/03), Instituto de Salud Carlos III, 28029 Madrid, Spain; 3 INCLIVA Biomedical Research Institute, Hospital Clínico, University of Valencia, 46010 Valencia, Spain

**Keywords:** pediatrics, obesity, cardiometabolic risk factors, precision nutrition, eating behavior, nutrigenetics, nutrigenomics, metabolomics, microbiota

## Abstract

The field of nutrition in early life, as an effective tool to prevent and treat chronic diseases, has attracted a large amount of interest over recent years. The vital roles of food products and nutrients on the body’s molecular mechanisms have been demonstrated. The knowledge of the mechanisms and the possibility of controlling them via what we eat has opened up the field of precision nutrition, which aims to set dietary strategies in order to improve health with the greatest effectiveness. However, this objective is achieved only if the genetic profile of individuals and their living conditions are also considered. The relevance of this topic is strengthened considering the importance of nutrition during childhood and the impact on the development of obesity. In fact, the prevalence of global childhood obesity has increased substantially from 1990 and has now reached epidemic proportions. The current narrative review presents recent research on precision nutrition and its role on the prevention and treatment of obesity during pediatric years, a novel and promising area of research.

## 1. Introduction

Nutrition is known to play one of the key roles in the prevention and treatment of non-communicable chronic diseases. Many of the molecular mechanisms through which nutrients affect the functioning of our bodies are now known. Promoting the correct functioning of these mechanisms through what we eat is the basis of precision nutrition. The goal now is to find personalized dietary strategies that improve people’s health.

Cardiometabolic diseases (CMDs) are the leading global cause of death, among which, obesity is the most frequent in childhood and adolescence [1]. Obesity, even during childhood, is a chronic disease with multifactorial etiology. Genetics, lifestyle factors, an unhealthy diet, sedentarism, and poor sleeping habits are some of its main causes, and all of them play an important role in its progression and the development of its comorbidities [2,3].

**Figure 1:**
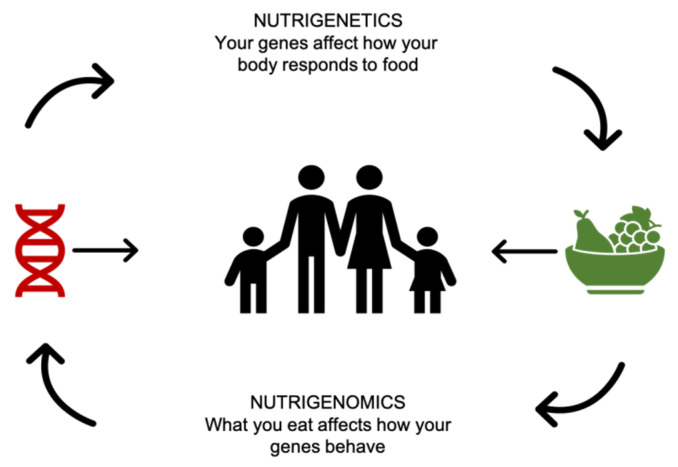
Nutrigenetics vs. nutrigenomics.

Physiological functions in the body can be modulated by nutrients because of their ability to interact with molecular mechanisms: “Nutritional genomics focuses on the interaction between bioactive food components and the genome, which includes nutrigenetics and nutrigenomics. The influence of nutrients on gene expression is called nutrigenomics, while the heterogeneous response of gene variants to nutrients, dietary components, and developing nutraceuticals is called nutrigenetics” [4] (Figure 1).

Herein, we highlight three of the hypotheses underpinning nutrigenetics and nutrigenomics [5]:

- “The health effects of nutrients and nutriomes (nutrient combinations) depend on inherited genetic variants that alter the uptake and metabolism of nutrients and/or the molecular interaction of enzymes with their nutrient cofactor and hence the activity of biochemical reactions.”
- “Nutrition may exert its impact on health outcomes by directly affecting the expression of genes in critical metabolic pathways and/or indirectly by affecting the incidence of genetic mutation at the base sequence or chromosomal level which, in turn, causes alterations in gene dosage and gene expression.”
- “Better health outcomes can be achieved if nutritional requirements are customized for each individual taking into consideration both his/her inherited and acquired genetic characteristics depending on life stage, dietary preferences, and health status.”

The objective pursued by precision nutrition is to design personalized feeding recommendations that allow for the treatment or prevention of CMDs [6]. For this purpose, it is not only based on genetic information, but also other components such as dietary habits, physical activity, the microbiota, and the metabolome.

### Purpose of Review

Nutrition plays a critical role during childhood and adolescence. Understanding the impact of nutrition in early life is essential for the development of future intervention strategies in order to modulate both immediate adult, offspring, grand offspring, and further phenotypes. Information on the potential of prescribing personalized nutrition is, however, scarce. Therefore, the aim of this narrative review is to provide an update on the evidence of the results of precision nutrition interventions during the early period of life, focusing primarily on its impact on obesity and cardiometabolic health. We focused our review on interventions (non-randomized pre-post intervention studies, clinical trials) or meta-analysis of interventions, mainly based on a personalized nutrition approach, in which their objective was to prevent or to treat obesity in infants and adolescents. We also included interventions during pregnancy due to the impact of a mother’s nutrition on their offspring. In order to structure the review, we followed the classification of precision nutrition interventions levels, proposed by Ordovás et al. [7].

## 2. Precision Nutrition Levels

Ordovás et al., propose two levels of personalization of nutrition advice. The first level incorporates a characterization of the subject’s behaviors and phenotype (such as adiposity) in order to develop a personalized nutritional advice. Interventions based in this level of personalization, were reviewed throughout the different pediatric stages (e.g., pregnancy, childhood, and adolescence). The second level of personalization builds on the first layer, while also considering different responses to foods and/or nutrients that are conditioned by genotypic or other biological characteristics (Table 1). 

### 2.1. Behavioral Level of Personalized Nutrition

The collection of an individual’s eating habits, behaviors, and phenotypic characteristics is the first level of personalization [7]. All this information combined is used to provide a dietary plan personalized to these characteristics and adapted to each period along the first stages of life.

#### 2.1.1. Pregnancy

Development during the intrauterine period determines life-lasting characteristics. Growing evidence suggests that childhood obesity and predisposition to metabolic disorders might be acquired as a result of erratic intrauterine attributes. In this aspect, the Developmental Origins of Health and Disease (DOHaD) hypothesis outlines the significant effects that the exposure to certain environmental stimuli since fetus development might have on an individual’s health. Even at the early stages of conception, the fetus, as a result of alterations that happen in its environment, is able to develop a series of predictive adaptations and adjustments in the homeostatic systems which will prepare it for a possible unfavorable postnatal setting. These adaptations, however, could have a negative impact as a result of an erratic interpretation of environmental changes. In this scenario, adaptations could be responsible for the development of diseases that might become chronic and even spread to future generations [8]. 

Therefore, pregnancy malnutrition impacts offspring adiposity and obesity, and its effects have been proven to spread to adult life independent of other lifestyle factors. Among the most referred to examples of undernutrition and its implications is the infamous Dutch famine case, in which these effects were observed in the offspring of women who had experienced severe caloric restriction during pregnancy [9]. In contrast, excess weight gain during pregnancy has also been related to metabolic and weight alterations in offspring. Additionally, the chances of developing pregnancy comorbidities such as gestational diabetes, preeclampsia, or cesarean birth are also enhanced [10]. 

Pregnancy weight control is, therefore, a key element in the health surveillance of mothers and their offspring. Numerous diet interventions have been shown to effectively control pregnancy weight gain, but their beneficial effects on offspring in the long term remain inconclusive according to a systematic review by Tanentsapf et al. [11].

Apparently, it is not only a matter of high energy intake, but also of the type of nutrients that women consume during pregnancy. Long-standing research suggests that what a woman eats during pregnancy not only influences children’s taste preferences across their lifetime [12,13], but may also influence children’s body composition and appetite. Few human research studies exist on this topic, although higher correlations have been shown between children’s protein and fat consumption at 10 years of age with their mother’s intake of these macronutrients during pregnancy than with her postnatal intake [14]. The relationship between saturated fat, and especially sugar dietary intake during pregnancy and offspring weight status was described by Murrin et al. [15].

A relationship between diet quality not only with an increased risk of developing obesity in offspring, but also with high blood pressure (BP) has been found. Data from two cohort studies in Scotland established in the 1950s [16,17] reported associations between a high-protein diet during pregnancy and high blood pressure in offspring in adulthood. Since then, many studies have shown similar evidence about the risk of a high-protein diet. A study performed among 965 women from the Danish Fetal Origins Cohort and their offspring after a 20-year follow-up showed an association between a higher mother´s intake of dietary protein during pregnancy with a slightly higher offspring BP [18]. 

Generating awareness of the risks associated with excess pregnancy adiposity could be an important factor in the establishment of healthy nutritional habits among parents that would prevent harmful events in offspring. Developing precision nutrition strategies for pregnant women could, therefore, be a high-impact preventive intervention.

#### 2.1.2. Lactation Period

Breastfeeding offers children and mothers unrivaled health benefits described since antiquity. The World Health Organization (WHO) suggests that breast milk is the “perfect food for the newborn and recommends all infants be exclusively breastfed up to six months of age, with continued breastfeeding along with appropriate complementary foods up to two years of age or beyond” [19]. 

Breastfeeding is shown to be a key component in the development of neurological, digestive and immune systems, and cellular health [20]. It is widely known that breastfeeding also delivers a protective effect against overweight [21]. A meta-analysis of observational studies suggested that this protective effect is dose-dependent, being more relevant the longer the duration of breastfeeding [22]. Another large meta-analysis demonstrated that the probability of developing obesity in children who had never been breastfed or who had been so during a period of less than six months was higher than for those having been breastfed for six months or longer [23]. In this regard, as demonstrated by previous studies [24], overweight or obese women during pregnancy are at a higher risk of not being able to breastfeed or having to interrupt it at an early stage. Therefore, the offspring become more vulnerable given the combination of an unfavorable fetal environment and the increased risk of developing cardiometabolic disease as a consequence of not benefiting from the properties of breastfeeding.

However, the physiopathological mechanism behind this protective effect is not fully understood, despite the large body of evidence supporting these benefits. Some hypotheses point toward the micronutrient and bioactive composition of breast milk [25,26]. On the one hand, it seems that the type of dietary constituent that the mother takes in while breastfeeding is an important part in modeling the future health of her offspring. Tahir et al. described how there was an inverse relationship between a higher maternal diet quality from pregnancy through three months postpartum with relative infant weight and adiposity from birth to six months and breastfeeding percentage at six months. Similarly, higher maternal diet quality during lactation was inversely related to infant fat mass at six months [27]. The exposure of infants to different flavors through breast milk can condition or shape their food preferences, both in the process of weaning and in later life [12]. In addition, some studies have demonstrated a lower likelihood of emptying a bottle or cup when compared to bottle-fed children [28], and that breastfeeding is associated with better self-regulation of eating by children [29], including better satiety responsiveness [30,31]. On the other hand, it is widely known that breast milk contains many bioactive hormones and peptides, with leptin being one of the most relevant. It is well documented that leptin is directly involved in the regulation of energy balance and food intake, and is crucial in the programming of metabolic pathways and infant appetite. This could explain that breastfeeding has a protective effect against childhood obesity compared to formula feeding [32], probably due to the lack of leptin in the later. Different studies have shown a clear association between leptin concentration in infants and in breast milk and infant body mass index (BMI) at two years of age [33]. They have established that moderate amounts of leptin supplied through breast milk seem to moderately protect infants from excess weight gain [34]. This benefit seems to last the longer the span of breastfeeding. 

The lower protein content of breast milk is also hypothesized to be a protective factor when it is being compared to formula milk [35,36]. Research with formula trials has demonstrated that enriched formulas lead to an increase in fat mass between five and eight years of age [37], and high protein content formulas have also been shown to pose a greater risk of obesity at school age [38,39,40]. The lower the amount of protein in the formula, down to certain limits, the greater the benefit. Comparisons between hydrolyzed and cow milk formulas have indicated faster weight gain and a doubled incidence in early rapid weight in the cow formula-fed group [41]. A modified formula with lower protein content has also been shown to have a protective effect on rapid weight gain [42].

Genome analysis has become a useful tool for the detection of individuals at a higher risk of developing obesity. Breastfeeding might have a direct impact on these patients. A recent study conducted by Wu et al. demonstrated that offering up to five months exclusive breastfeeding to children with a higher genetic risk obtained through an obesity-specific genetic risk score implied a substantial decrease in BMI [19]. This study proposed that breastfeeding is a key candidate intervention to reduce overweight risks in predisposed patients, since it demonstrated direct impact from the first moments of life. Detecting those people who carry alleles related to obesity, and then carrying out interventions for the promotion of breastfeeding in this group of people, should become a priority. 

Lastly, during the lactation period, weaning is crucial, since caregiver feeding behaviors could be an obesogenic influence on child eating behaviors [43]. Non-directive strategies such as repeatedly offering foods [44] and having caregivers portray eating the food with enjoyment have been demonstrated to increase the consumption of a given food and to support children’s liking for a wider variety of healthy foods, and may help maintain responsivity in the feeding environment [45,46,47]. However, pressure to eat has been associated with an impaired ability to self-regulate eating behaviors in preschool and poorer energy compensation in childhood [48]. Taken together, this evidence suggests a bidirectionality between child eating behaviors and adiposity, as well as caregiver feeding behaviors [49,50]. Therefore, interventions should also focus on how parents should feed their children and maintain healthy habits in the family from birth. 

Taking all of this scientific evidence into account, it seems that there is no better precision nutrition during the lactation period than breastfeeding, without ignoring the effect that caregivers have on children’s relationship with food.

#### 2.1.3. Childhood and Adolescence

The treatment of obesity is based on changes in lifestyle, mainly eating habits and physical activity. A meta-analysis including 3436 adults in 16 randomized controlled trials concluded that the Mediterranean diet, especially if the diet is energy-reduced and accompanied by physical activity, favors greater weight loss than a control diet [51]. According to the evidence obtained in this and other studies, vegetable-based diets such as the Mediterranean diet are the most appropriate for treating obesity [52]. However, there are also studies that show greater weight loss in protein-rich diets [53]. Another aspect is the quality of fats. Increasing the consumption of omega-3 or -6 polyunsaturated fatty acids happens to improve plasma lipid levels and on the other hand lowering the risk of cardiovascular events [54,55]. However, it appears that losing weight does not depend on macronutrient composition when comparing the effect of different low-calorie diets in adults, as shown in a review of 48 studies [56].

There are also discrepancies in the consensus on the best dietary strategy for treating obesity in children and adolescents [57]. It has been shown that low-carb or low-glycemic diets to have the same effect as standard portion-controlled diets [58]. Nevertheless, children’s adherence to modified carbohydrate diets in the long term may be low. In children and adolescents, semi-structured dietary approaches are best used to support children and their families to select healthier food groups and decrease portion sizes [59].

In a systematic review including 14 clinical trials conducted in children between 6 and 18 years old, the efficacy of seven interventions based on low-fat diets was compared against two isocaloric and five ad libitum low-carb diets [60]. This study showed that the weight status of participants improved by implementing a reduced-energy diet, irrespectively of macronutrient distribution. Moreover, the authors pointed out that the distribution of macronutrients in the diet could be tailored in each case according to the presence or not of comorbidities. For example, in the case of obesity associated with diabetes or insulin resistance, low-carbohydrate diets were chosen. However, they reported that more studies are needed to better define these personalized dietary guidelines.

All of this research suggests that it is the energy content and not the macronutrient composition of a diet playing the key role in weight loss. However, is this a consequence of the fact that, considering the complexity of obesity, we are not personalizing the type of nutrition enough?

Recently, the American Association of Clinical Endocrinologists (AACE) and the European Association for the Study of Obesity (EASO) proposed the use of a new diagnostic term in adults, namely, “adiposity-based chronic disease” (ABCD) [61,62]. Obesity is considered “adiposity-based” because the disease is primarily a result of abnormalities in the mass, distribution, and/or function of adipose tissue. A new coding system was proposed addressing the pathophysiological heterogeneity of obesity [63]. This code is based on three dimensions. First, etiology, including two mechanistic categories: (1) multifactorial disease (the majority of patients) and (2) specific identifiable factor obesity. Second, the degree of adiposity. Third, health risk, taking into account the presence or absence of comorbidities. This proposal constitutes an advancement because obesity is recognized as a heterogeneous disease with complex pathophysiology, and it includes a dimension revealing its impact on health [61].

In order to have enough information to tailor an adequate treatment, the diagnostic procedures of an obese child may include family history, prenatal factors, feeding history, sleep duration and matters, exercise and the time spent looking at screens, when and where meals are taken, bullying or social isolation, motivation and the capacity to make modifications within a family, and the family’s cultural expectations and financial limitations. Laboratory tests and studies are considered according to age, BMI percentile, and the presence of risk factors. Children presenting with growth deceleration, symptoms of hypo-hyperthyroidism or other endocrinopathies, sustained hypertension, symptoms of type 2 diabetes (T2DM), a family history of early cardiovascular disease, hirsutism, snoring, and/or daytime sleepiness need further work-up. Therefore, obesity is increasingly seen as a complex disease with different etiologies. As a consequence, the need for an individual nutritional approach instead of “one-size-fits-all” recommendations is growing.

Personalized nutrition-based interventions in obese children and adolescents are scarce in the literature. Recently, Lim et al. reported the results of a 16-week long lasting evidence-based customized nutritional intervention in 103 subjects [64]. For each patient, an intervention was implemented following a nutrition care process (NCP) model. Both at baseline and follow-up, the sociodemographic and anthropometric data, health and dietary behavior, and dietary intake of the participants were assessed. All participants engaged in 30-min nutritional sessions held each month. Four steps were used to implement the NCP: (1) Nutrition assessment, (2) diagnosis, (3) intervention, and (4) monitoring/evaluation. Nutritional diagnosis was performed on the basis of three areas: Intake (amount of food or nutrient), clinical factors (nutritional problems linked to medical or physical circumstances), and behavioral–environmental factors (attitudes, knowledge, beliefs, food access and safety). On the basis of this information, a personalized intervention was planned for each subject. The study has some limitations, such as not having a control group and the short duration of the follow-up. Moreover, although participants globally improved in body composition, macronutrient intake, and nutritional behavior, the results were not superior to those of other non-tailored interventions.

Another area of study is the relationship between obesity and eating disorders (EDs), because the presence of an eating disorder can suppose a radical change in the therapeutic approach of the patient. Overweight and obesity knowingly increases the risk of developing EDs [65]. However, the assessment and management of EDs caused by or being a consequence of obesity are not contemplated with sufficient caution in children and adolescents [66]. Developing effective tools to properly diagnose these disorders and designing specific therapies that combine nutritional, psychological, behavioral, and/or pharmacological treatment in children and adolescents can be a further step in the development of personalized therapeutic strategies [67].

A systematic review conducted following preferred reporting items for systematic reviews and meta-analyses (PRISMA) guidelines tried to answer the question: “Is multidisciplinary treatment (MT) effective on eating disorder symptoms in children with obesity?” [68]. Concerning psychology, all studies except one included support group therapy. The review showed that MT not only decreased the BMI Z-score (zBMI), but also positively influenced external eating, disinhibition of control, and emotional eating. They concluded that MT might be influenced by eating behavior, and participants learned to face emotional stress and external stimuli. Nevertheless, a recently published study identified four patterns of ED pathology [69]: only loss of control, shape and weight concerns (SWCs), low ED pathology, and high ED pathology. After the intervention, all groups shared an important reduction of their zBMI; however, no clinically significant weight loss was reached by those with the highest ED pathology. The authors concluded that further research is recommended in order to identify strategies modifying treatment for children who suffer of both obesity and high ED pathology to improve weight loss success.

More research is needed in this field in order to be able to offer obese children and adolescents an intervention that is adjusted to the etiology of the disease, the degree of obesity, and the comorbidity that it presents, in addition to the fact that it must be viable considering the patient’s sociocultural environment. Behavioral level interventions included in the review are summarized in Table 2.

### 2.2. Biological Levels of Personalized Nutrition

With the information obtained from the regular diagnostic work with an obese patient, it does not seem that we can identify the type of nutritional intervention that is most appropriate for each patient [70]. Therefore, the identification of biomarkers that would allow us to predict the response of an individual to a given diet or lifestyle advice is of enormous value. This is the current challenge in the field of precision nutrition [71].

#### 2.2.1. Predictive Biomarkers

There is enormous variability in the response of obese children and adolescents to dietary interventions. Currently, research continues in order to develop pretreatment prognostic markers that can identify patients who may benefit from a particular dietary weight loss intervention. However, some factors, such as the differences in dietary adherence, psychological, socioeconomic factors, and other unknown ones (e.g., genes, epigenetics, and pollutants), make the identification of these biomarkers a scientific challenge.

However, recent discoveries have identified that alterations in the mechanisms linking glucose metabolism to appetite control could be involved in the big variability in weight loss observed in obese patients prescribed the same diet. In adults, studies conducted by Astrup and Hjorth [72] suggested that “individual weight loss responsiveness to diets depends on glucose metabolic traits that, clinically, can be characterized by fasting glucose and insulin levels for each patient before initiation of treatment. Based on these measurements, an optimal diet composition can be tailored to enhance satiety, adherence, and weight loss.” They hypothesized that carbohydrate-rich meals may be very satiating in insulin-sensitive overweight (type A), less so in more insulin-resistant obese prediabetics (type B), and even less so in obese individuals with T2DM (type C). This hypothesis is based on reanalysis by these authors of some of the largest clinical trials (e.g., PREDIMED, the NUGENOB study, and the Diet, Obesity, and Genes (DiOGenes) trial) that seemed to indicate that the macronutrient composition of diets does not really matter. They showed that, when reclassifying each participant into one of these conditions (types A, B, or C), weight loss responsiveness and best-fit diet are completely different [73,74]. These studies showed that prescribing personalized weight-control diets on the basis of pretreatment glycemic (and insulinemic) status is a promising field of research.

A review conducted by Lister et al. [75] identified specific dietary strategies that can reduce the risk of T2DM development in obese youths. The authors concluded that, in addition to weight loss, a reduced carbohydrate diet may optimize improvements in T2DM risk factors, including hepatic steatosis, insulin resistance, and hyperglycemia. The DiOGenes study, included in this review is, to date, the largest study aimed at examining the effect of modifying the glycemic index (GI) and protein content of a diet on weight status and cardiometabolic outcomes in children. Participants were randomized as a family unit to one of five ad libitum diets: low-protein and low-GI, low-protein and high-GI, high-protein and low-GI, high-protein and high-GI, and control diet (medium protein content and no instructions on GI) [76]. The results of this study showed that, among children, neither GI nor protein ad libitum diets had an isolated effect on body composition. However, the low-protein and high-GI combination increased body fat, while the high-protein and low-GI combination was protective against obesity [77]. Although these types of diet have some inconveniences, such as worse adherence, fatigue-inducing physical activity, and the risk of reducing the intake of fiber and phytochemicals if vegetable intake is not increased, they could be considered as an option to tailor nutrition in obese children and adolescents at greater risk of developing T2DM.

#### 2.2.2. Genetics

It is estimated that monogenic diseases with Mendelian inheritance represent approximately 5% of non-syndromic cases of obesity, including mutations in the leptin receptor and *BDNF*, *MC4R*, *MC3R*, *PCSK1*, *PCSK2*, *POMC*, *PPARG*, *SIM1*, and *TRKB* genes, among others. However, obesity is generally considered to be a multifactorial disease with high heritability (50–75%), probably higher in cases of early onset. In children who develop severe obesity before 5 y.o., genetic causes should be taken into account. These children may present clinical features such as short stature, developmental delay, hyperphagia, or dysmorphic facies. Work-up in these patients involves DNA methylation studies, exome sequencing, and karyotyping for syndromes such as Albright’s hereditary osteodystrophy, Alstrom, Bardet–Biedl, Cohen syndrome, Fragile X, Prader Willi, congenital leptin deficiency, *POMC* deficiency and *MC4R* deficiency. Nevertheless, other genetic causes of obesity not associated with described syndromes could also be contributors [78]. 

The association of body weight and genetic loci has been identified in hypothesis-driven candidate gene studies [79]. In these studies, the investigated genes were mainly chosen for their role in regulating food intake. For example, the transmembrane protein 18 (*TMEM18*) gene and melanocortin-4 receptor (*MC4R*) have a function in regulating food intake; fatty acid-binding protein 2 (*FABP2*) is involved in lipid metabolism; peroxisome proliferator-activated receptor-gamma (*PPARG*) and the fat mass- and obesity-associated (*FTO*) locus have a role in adipocyte differentiation. Specifically, almost 100 loci were identified, accounting for approximately 2.7% of the variation in BMI, of which the *FTO*, *MC4R*, and *TMEM18* genes showed the strongest associations. To date, the gene with the strongest relation on body fat is the *FTO* locus; however, the biological functions of most of these loci remain unclear [80]. Understanding the genetic mechanism behind obesity will likely help to develop future tailored interventions for the prevention and treatment of childhood obesity.

On the basis of these facts, it could be hypothesized that subjects with these non-favorable mutations respond worse to weight-loss interventions. Nevertheless, the results of a systematic review and meta-analysis including 9563 adults based on the association between the *FTO* locus gene and weight loss [81] showed that after dietary intervention, carriers of the *FTO* locus-risk allele of single-nucleotide polymorphisms (SNP) rs9939609 achieved similar weight loss compared to that of non-risk allele carriers. Recently, the results of the randomized controlled trial “Diet Intervention Examining the Factors Interacting with Treatment Success” (DIETFITS) have been published. These results again showed, in 609 overweight adults, that weight reduction is independent of genotypes [82]. 

Data on children are scarce. One of the studies with a larger number of recruited subjects (684; 280 boys and 404 girls) assessed how the *NYD-SP18* and *TMEM18* variants affected the efficacy of an intensive one-month inpatient diet and physical activity weight-reduction program [83]. Neither *NYD-SP18* nor the *TMEM18* variant was associated with changes in anthropometric measurements after the lifestyle intervention. Few studies on similar topics, including *TMEM18* polymorphism, have been published. Hiney et al. [84] analyzed 282 obese children and found no evidence for the effects of this gene on weight loss or regain one year after an intervention, and similar results were observed in an analysis of 400 children/adolescents [85]. However, a Spanish study analyzed 168 overweight/obese adolescents for the contribution of nine obesity-related polymorphisms, as well as genetic predisposition scores (GPSs) on the changes in body composition and cardiometabolic risk factors in a three-month intervention, on the basis of a personalized diet and a physical activity program [86]. They included *FTO*, *MC4R*, *PPARG*, and *TMEM18* SNPs. The authors detected, after adjusting for baseline BMI standard deviation score that subjects with a higher GPS had smaller improvements in metabolic profile and a worse response to physical activity compared to those subjects with a lower GPS. The limitations of this study were that the sample was small and that it included just three months of follow-up. Nevertheless, these findings were not confirmed by a larger intervention including 920 overweight and obese subjects from the Danish Childhood Obesity Biobank [87]. A genetic risk score (GRS) comprising 15 SNPs associated with childhood obesity was assessed. Authors demonstrate that these genetic variants did not predict the response to lifestyle changes. It could be concluded that a high genetic predisposition to overweight during childhood in fact had no influence on whether the children reacted to lifestyle intervention compared to children with low genetic predisposition to overweight, so these genetic risk scores have not demonstrated being a useful tool to tailor nutritional interventions.

#### 2.2.3. Metabolomics

Metabolomics studies are essential to understand the effect of food on an individual’s health. The identification of food-derived biomarkers makes it possible to know how different subjects distinctly metabolize the same food, and how such food metabolites may influence health outcomes. In this regard, obtaining reference values for food metabolites is necessary. Recently, a study established a reference dataset of mainly healthy subjects [88] and its results allowed for differentiating normal metabolomes between different groups (e.g., men/women and elderly/young).

Another useful field of metabolomics is its potential in determining the pattern of food consumption of an individual [89]. A major challenge of precision nutrition remains the objective measurement of adherence to a dietary pattern. In this sense, the spectroscopic profiling of urine was validated for the objective measurement of an overall dietary pattern [90]. This technique is being used to characterize eating patterns in an ongoing study in which our group participated, with the main aim of exploring the individual eating/behavioral patterns, parental styles, life events, cognitive styles, and biological-endocrine factors associated with the development of abnormal eating behaviors, EDs, or obesity (i.e., the EAT4HEALTHYLIFE Project) [91].

#### 2.2.4. Microbiota

Microbiotainteract directly with the host’s cells through a bidirectional equilibrium relationship, and impact multiple facets of its physiological and biochemical pathways involved in immunity and energy homeostasis. Microbiota are considered one of the most densely populated ecosystems with a vast gene pool, sometimes referred to as our “second genome,” with the capacity of being malleable and significantly varying from host to host. 

As mentioned, variations in our genome have a direct impact on the metabolism of nutrients between individuals. The gut microbiome also appears to significantly impact these variations. In this regard, precision microbiomics can be described as “the use of the gut microbiome as a biomarker to predict the effect of specific dietary components on host health and the use of these data to design precision diets and interventions that ensure optimal health” [92]. This approach sets a much more complex scenario when compared to the one-fit-for-all diet approaches that appear to perform poorly [93]. 

The understanding of the paths in which the microbiome is related to the substantial changes in inter-individual responses to lifestyle and diet interventions is a key element for the development of new tools that help enhance the potential of individual microbiomes as a source of human variation modulating dietary responses [94]. Diet shapes microbiota composition [95]; however, microbiota vary their interaction within the same diet depending on their composition and host characteristics [94]. Data on how a diet directly shapes the microbiome and how this affects body composition are complex and not completely understood.

In a review by Biesiekierski et al., microbiota are described as a predictive tool regarding host behavior toward diet interventions [96]. Although there are data that demonstrate that gut microbiota composition and response to diet are linked, this review pointed that only a relatively small number of interventions show health benefits, and current data are inconsistent to establish whether certain strains can predict the response that each individual has to a certain dietary intervention.

However, some authors have explained that this analysis between the response of a population group as described in randomized trials and a specific dietary pattern may result in an oversimplified vision of the correlations between diet and gut microbiota [97] without considering the diversity within an individual. When analyzing the response to a certain intervention, it seems important to know the status of the basal microbiome of each individual, since it determines the response. Studies have shown that the initial microbiota of the host at the time of initiating a dietary intervention influence the response to it and the possible changes that may occur both in the host and in their own microbiota [98]. 

It is now becoming apparent that long-term healthy dietary patterns—in particular, habitual fiber intake—are crucial for highly diverse gut microbiota responsiveness to specific interventions. Low-diversity gut microbiota can benefit from specific dietary interventions if they contain even minimal amounts of responding and effector microorganisms [99]. Patients whose poor dietary practices or very low dietary fiber intake are prolonged during long periods of time could experience the loss of beneficial microbial lineages which would, in turn, make any dietary intervention fail [100]. There are some studies focused on promoting favorable changes in microbiota of obese children and adolescents using prebiotic supplementation [101] or through food (mainly whole grain) [102].

Understanding the relevance of these interventions on gut microbiota and their impact on health is in an early phase, and evidence-based scientific validation in children is scarce, but the use of the microbiome as a biomarker of response to certain interventions is encouraging. In the aforementioned review by Biesiekierski et al. [103], the results may not have been significant because the immense variability among different study participants was not considered. Methodological consistency between studies is also improvable. As far as we know, no such interventions have been performed on obese children or adolescents. Whole microbiome next-generation sequencing performs differently when compared to selective sequencing combined with specimen cultures.

Given the vast amount of information that can be obtained from a whole microbiome, its understanding and, therefore, the knowledge that can be applied to dietary decision making are still limited. Big data analysis is also experimenting with exponential improvements, and modifies and impacts obtained conclusions from previous data pools. There are already studies that use microbiome information for better approaches to machine learning and developing personally tailored diet interventions [104]. The first was carried out by Zeevi et al. [105], who developed an innovative algorithm to predict postprandial glucose responses according to anthropometry, blood test results, and the microbiome composition of 1000 people. In a study by Korem et al., the impact of different types of bread on health benefits was found to be determined by the gut microbiome as opposed to its ingredients [106]. Biological level interventions included in the review are summarized in Table 3.

## 3. Future Directions

The prevalence of obesity and the associated cardiometabolic risk factors in children and adolescents has been globally increasing since 1990. Nowadays, there is growing evidence from basic nutritional science about the importance of dietary advice, and it is considered one of the main challenges of clinical nutrition. Moreover, tailored nutrition represents a promising approach to prevent and manage obesity. A concerted effort between clinical and basic science researchers is needed in order to establish a comprehensive framework to allow the implementation of these new findings to adequately apply novel and personalized dietary advice.

## Figures and Tables

**Table 1:** Levels of personalization of nutrition-based interventions in the prevention and treatment of obesity throughout the different pediatric stages.

Level of Precision Nutrition		Pediatric Stage		
1. Behavioral Level		1.1. Pregnancy [8,9,10,11,12,13,14,15,16,17,18]	1.2. Lactation period [19,20,21,22,23,24,25,26,27,28,29,30,31,32,33,34,35,36,37,38,39,40,41,42,43,44,45,46,47,48,49,50]	1.3. Childhood and adolescence [51,52,53,54,55,56,57,58,59,60,61,62,63,64,65,66,67,68,69]
		First 1000 days		
2. Biological Levels	2.1. Biomarkers [70,71,72,73,74,75,76,77]			
	2.2. Genetics [78,79,80,81,82,83,84,85,86,87]			
	2.3. Metabolomics [88,89,90,91]			
	2.4. Microbiota [92,93,94,95,96,97,98,99,100,101,102,103,104,105,106]			

[xx] References of articles included in each subheading.

**Table 2:** Summary of behavioral level interventions included in the review.

Authors/Reference	Type of Study	Subjects/Studies	Clinical Condition	Type of Intervention	Outcomes: Primary (1st) and Secondary (2nd)	Key Findings
**Pregnancy**						
Tanentsapf et al.; BMCpregnancy and Child-birth. 2011 [11].	Mct	N: 1434 13: RCT (10) quasi-RCT (3) pregnant women	H, Ow, Ob > 18 years	D or DI vs. UC (*n* = 9) D vs. DI vs. UC (*n* = 1) D + placebo vs. UC + placebo (*n* = 1). D vs. UC (*n* = 1) DI vs. US (*n* = 1)	1st: % of women excess GWG 2nd: maternal (GD, PRE, CS, WG) & infant (BW, PTB, GA)	Dietary advice in pregnancy can decrease GWG and postpartum weight retention
**Lactation period**						
Singhal et al., Am. J. Clin. Nutr. 2010 [37].	Mct	Study 1 (*n* = 299) Study 2 (*n* = 246) Follow up from birth to 5–8 years	>37 weeks Study 1: BW < 10th pct. Study 2: BW < 20th pct	STF vs. NEF	1st: childhood adiposity (fat mass)	NEF increased body fat mass in later childhood independent of other confounding factors
Weber et al., Am. J. Clin. Nutr. 2014 [39].	RCT	*n* = 755 (HPF 256, LPF 262, BF 237) 6 y follow-up	H term infants	Intervention group: HPF vs. LPFReference group BF	1st: BMI2nd: weight, height, and obesity	HPF implies higher risk of obesity in childhood
Mennella et al., Pediatrics 2011 [41].	RCT	*n* = 56 (CMF 32, PHF 24) 7 mo follow up	H term infants	CMF vs. PHF	1st: trajectories growth measures2nd: F acceptance	CMF greater weight gain velocity
Kouwenhoven, Am. J. Clin. Nutr. 2020 [42].	RCT	*n* = 235 (mLPF 88, CTF 82, BF 65) 6 mo follow up	H term infants	Intervention group: mLPF vs. CTFReference group BF	1st: daily WG2nd: F intake, growth, BC, BT, AEs	mLPF has protective effect on rapid weight gain
**Childhood and adolescence**						
Kirk et al., J Pediatr. 2012 [58].	RCT	102 (7–12 years) 43% male	Ob BMI Z-score range (1.60–2.65)	3-month intervention randomly assigned to LC, RGL, or PC diet+exerc.	1st: BMI z-score, WC, %BF and diet adherence 2nd: clinical metabolic parameters	LC and RGL as effective as PC at at 3, 6 and 12 months, but significantly lower adherence to LC
Go et al. Nutr Rev. 2014 [60].	Mct	14: RCT (12) quasi-RCT (2) <18 years 8 weeks-2 years follow up	Ow + Ob	LC vs. PC (*n* = 7) IP vs. SP (*n* = 6) IF vs. SF (*n* = 1)	1st: BMI, BMI z-score, %BF 2nd: CMB parameters	Achieved improvements in weight status irrespective of macronutrient distribution of a reduced-energy diet
Kim et al. Nutr Res Pract. 2020 [64].	RCT	103 (7–12 years) 61% male	Ob 67% moderate 33% severe	16-week nutritional intervention tailored for each subject based subject’s specific nutrition diagnosis.	1st: BMI z-score, WC, %BF 2nd: stage of change and diet adherence	Intervention improved body composition and decreased calorie intake in adherent subjects
De Giuseppe et al. Front. Pediatr. 2019 [68].	Mct	9: RCT (3), non-RCT (1), ITS (5) 6 to 18 years	Ow/Ob + ED	Heterogeneous MT All: support group therapy + CBT	1st: ED symptoms and MT features 2nd: BMI	MTs reduced BMI positive short- and long-term impact of MTs on ED symptoms

AEs: adverse events, BC: body composition, BF: breastfed, %BF: body fat percentage, BMI: body mass index, BW: birth weight, BT: blood test, CBT: cognitive behavioral therapy, CMB: cardiometabolic, CMF: cow’s milk formula, CS: cesarean section, CTF: control formula, D: diet, DI: diet and intervention, ED: eating disorder, F: formula, GA: gestational age, GD: gestational diabetes, GWG: gestational weight gain, H: healthy, HPF: high-protein formula, IF: increased fat diet, IP: increased protein diet, ITS: interrupted time series without comparison group, LC: low-carbohydrate diet, LPF: low-protein formula, Mct: meta-analysis of clinical trials, mLPF: modified low-protein formula, MT: multidisciplinary treatment, NEF: nutrient-enriched formula, Ob: obese, Ow: overweight, PHF: protein hydrolyzed formula, PC: standard portion-controlled diet, PRE: preclampsia, PTB: preterm birth, RCT: randomized clinical trial, RGL: reduced glycemic load diet, SF: standard-fat diet, SP: standard protein diet, STF: standard-term formula, UC: usual care, WG: weight gain.

**Table 3:** Summary of pediatric biological level interventions included in the review.

Authors/Reference	Type of Study	Subjects/Studies	Clinical Condition	Type of Intervention	Outcomes: Primary (1st) and Secondary (2nd)	Key Findings
**Predictive Biomarkers**						
Papadaki et al., Peds, 2010 [71].	RCT	Baseline 827 (5–18 years) Completers, 26 weeks: 465 (43% male)	Healthy children from selected families	Ad libitum diets 1–5: LP/LGI *n* = 102.Do: 37% LP/HGI *n* = 87. Do: 48% HP/HGI *n* = 92. Do: 42% HP/HGI *n* = 96. Do: 39% PC *n* = 88. Do: 42%	1st: BMI z-score, %BF 2nd: %OW-Ob and waist/hip ratio.	No effect on body composition was observed by GI nor protein isolated. However, LP/HGI increased body fat, while HP/LGI protected against obesity
**Genetics**						
Zlatohlaveketal, Med Sci Monit, 2018 [78].	Pre-post	684 (12–14 years) 41% male	Ob + Ow	One-month inpatient intensive lifestyle intervention: nutritional + physical activity *TMEM*-18 Vs *NYD-SP18* gene variants	1st: BMI z-score, waist, hip, abdominal skinfold 2nd: genotype	No significant differences in BMI changes among gene variants
Hiney et al. J Pediatr Endocr Met 2013, [84].	Pre-post	282 (8–13 years) 47% male	Ob + Ow 14% over-weight, 86% obese	1y. lifestyle intervention. Reevaluation 1y. after end intervention. Evaluate regain weight taking into account genes: *NEGR1*, *TNKS*, *SDCCAG8*, *FTO*, *MC4R*, *TMEM18*, *PTER*, *MTCH2*, *SH2B1*, *MAF*, *NPC1*, and *KCTD15*	1st: BMI SDS2nd: genotype	None of the SNPs including were related to weight regain after 1y.
Moleres et al. Jpeds. 2012, [86].	Pre-post	168 (12–16 years)	Ob + Ow 49% over-weight, 51% obese	6–24 months lifestyle intervention Subjects were genotyped for 9 obesity-related SNPs in the *FTO*, *MC4R*, *TMEM18*, *IL6*, *PPARG*, and *ADIPQ* genes and a GPS was calculated	1st: BMI, BMI z-score, %BF 2nd: genotype, CMB parameters	The GPS had relationship with BMI-SDS and fat mass both at baseline and after a 3-month intervention.
Hollensted et al. Obesity (2018) [87].	Pre-post	1674 (9–15 years)	Control group *n* = 754/ Ob + Ow group *n* = 920	3 months lifestyle intervention Subjects were genotyped for 15 obesity-related SNPs and a GPS was calculated	1st: BMI, BMI z-score, %BF 2nd: genotype, CMB parameters	The GPS had relationship with BMI-SDS at baseline in both groups, but did not influence response to intervention.
**Metabolomics**						
Fernandez Aranda et al., (study in progress) 2018-20 [91].	Pre-post	50 (8–10 years -prepubertal- and 12–16 years -post pubertal-)	Ob + -EDs	12-month lifestyle intervention	1st: BMI, BMI z-score, %B, EDs 2nd: Endocannabinoid profile	Hypothesis: relation among obesity, EDs and Biochemical, hormonal and Endocannabinoids blood levels. Metabolomic-based diet pattern
**Microbiota**						
Nicolucci et al. J.gastro. 2017. [101].	RCT	42 (7–12 years) 57% male	Ob + Ow 67%	16-week once daily supplementation OI group *n* = 22 vs. M group *n* = 20	1st: BMI z-score, %BF 2nd: effect of prebiotic supplementation on gut microbiota, FBAs, CMB parameters	OI significant decrease of BMI, BF, IL6, TGs vs. M. OI increases *Bifidobacterium* spp. and decreases *Bacteroides vulgatum*
Zhang a,1,et al. J.ebiom. 2015 [102].	Pre-post	40 (3–16 years)	PWS children *n* = 19 Ob children *n* = 21	Nutritional in-hospital intervention (ob 30 days, PWS 90 days) WTP diet.	1st: weight loss 2ng: changes gut microbiota	MTs reduced BMI positive short- and long-term impact of MTs on ED symptoms

ADIPQ: Human adiponectin gene, EDs: eating disorders, Do: drop-out rate, FBAs: fecal bile acids, FTO: fat mass and obesity-associated gene, GI: glycemic index, GPS: genetic predisposition score, HGI: high glycemic index diet, HP: high protein diet, IL6: Interleukin 6 gene *, KCTD15: potassium channel tetramerization domain containing 15 genes* LGI: low glycemic index diet, LP: low-protein diet, M: maltodextrin placebo, *MAF: v-maf musculoaponeurotic fibrosarcoma oncogene homolog (avian) gene*, MC4R: melanocortin 4 receptor gene, MTCH2: mitochondrial carrier 2 gene, NEGR1: Neuronal growth regulator 1 gene, *NPC1: Niemann-Pick disease, type C1, gene;* OI: oligofructose-enriched inulin, PC: standard portion-controlled diet, PPARG: Peroxisome proliferator-activated receptor gamma gene, PTER: phosphotriesterase related gene, PWS: Prader–Willi Syndrome, Pre-post: non-randomized pre-post intervention studies, SDS: standard deviation score, SDCCAG8: serologically defined colon cancer antigen 8 gene, SH2B1, SH2B: adaptor protein 1 gene, SNPs: single nucleotide polymorphisms, TMEM18: transmembrane protein 18 gene, TNKS: tankyrase gene, WTP: diet based on whole grains, traditional Chinese medicinal foods and prebiotics.
